# Computational Analysis of Pathogenetic Pathways in Alzheimer’s Disease and Prediction of Potential Therapeutic Drugs

**DOI:** 10.3390/brainsci12070827

**Published:** 2022-06-24

**Authors:** Maria Cristina Petralia, Katia Mangano, Maria Catena Quattropani, Vittorio Lenzo, Ferdinando Nicoletti, Paolo Fagone

**Affiliations:** 1 Department of Clinical and Experimental Medicine, University of Messina, 98122 Messina, Italy; m.cristinapetralia@gmail.com; 2 Department of Biomedical and Biotechnological Sciences, University of Catania, Via S. Sofia 97, 95123 Catania, Italy; kmangano@unict.it (K.M.); paolofagone@yahoo.it (P.F.); 3 Department of Educational Sciences, University of Catania, 95124 Catania, Italy; maria.quattropani@unict.it; 4 Department of Social and Educational Sciences of the Mediterranean Area, University for Foreigners “Dante Alighieri” of Reggio Calabria, 89125 Reggio Calabria, Italy; v.lenzo@unidarc.it

**Keywords:** Alzheimer’s disease, dementia, therapeutic targets, in silico pharmacology

## Abstract

Background. Alzheimer’s disease (AD) is a chronic and progressive neurodegenerative disease which affects more than 50 million patients and represents 60–80% of all cases of dementia. Mutations in the APP gene, mostly affecting the γ-secretase site of cleavage and presenilin mutations, have been identified in inherited forms of AD. Methods. In the present study, we performed a meta-analysis of the transcriptional signatures that characterize two familial AD mutations (APP^V7171F^ and PSEN1^M146V^) in order to characterize the common altered biomolecular pathways affected by these mutations. Next, an anti-signature perturbation analysis was performed using the AD meta-signature and the drug meta-signatures obtained from the L1000 database, using cosine similarity as distance metrics. Results. Overall, the meta-analysis identified 1479 differentially expressed genes (DEGs), 684 downregulated genes, and 795 upregulated genes. Additionally, we found 14 drugs with a significant anti-similarity to the AD signature, with the top five drugs being naftifine, moricizine, ketoconazole, perindopril, and fexofenadine. Conclusions. This study aimed to integrate the transcriptional profiles associated with common familial AD mutations in neurons in order to characterize the pathogenetic mechanisms involved in AD and to find more effective drugs for AD.

## 1. Introduction

Alzheimer’s disease (AD) is a chronic and progressive neurodegenerative disease which affects more than 50 million patients and represents 60–80% of all cases of dementia [1]. The pathological feature of AD is the accumulation of extracellular amyloid-β (Aβ) plaques and intracellular neurofibrillary tangles (NFTs) in the brain, leading to the loss of neurons and synapses, and consequently to cognitive impairment and dementia [2,3].

Amyloid precursor protein (APP) is a type I transmembrane protein that is proteolytically cleaved by secretases to give rise to the Aβ peptides. Cleavage of APP by α-, β-, δ-, and η-secretases results in the secretion of the large extracellular APP domain [4]. On the other hand, γ-secretase gradually cleaves APP within its transmembrane domain, thereby releasing 37–43 residue-long secreted Aβ peptides [4]. The γ-secretase consists of four subunits—the proteolytically active subunit presenilin (PSEN) and three non-proteolytic subunits (nicastrin, anterior pharynx defective 1, and presenilin enhancer 2)—necessary for the assembly and stabilization of the quaternary structure [4]. AD mutations in the APP gene mostly affect the γ-secretase site of cleavage. Furthermore, presenilin mutations were identified in dominantly inherited forms of AD. The result of these mutations is the generation of abnormal Aβ peptides, which aggregate and constitute the amyloid plaques [4].

In the present study, we performed a meta-analysis of the transcriptional signatures that characterize two familial AD mutations (APP^V7171F^ and PSEN1^M146V^) in a neuron model. The causal role of mutations in the APP and PSEN1 genes has long been known, but their precise consequences at the cellular level remain incompletely characterized, which makes the identification of effective novel therapeutic strategies challenging.

**Figure 1:**
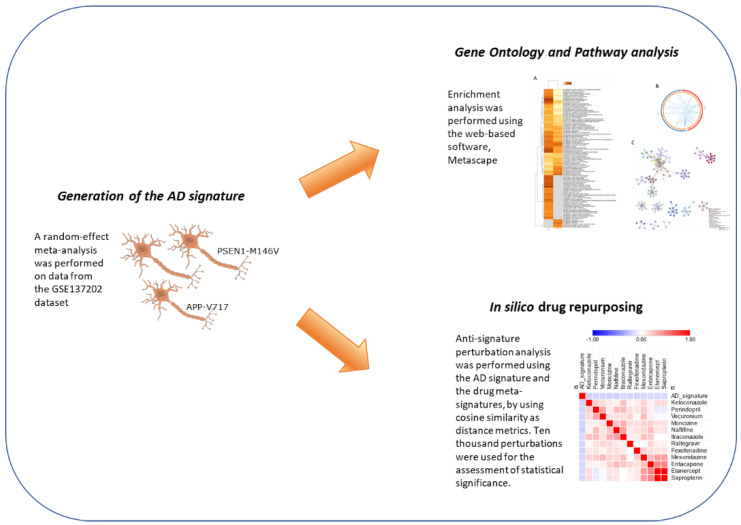
Experimental layout.

Next, the identified AD-related gene expression pattern was used to predict a number of drugs, which may potentially be able to revert the transcriptional changes associated with the AD pathology (Figure 1 shows the overall layout of the experimental design). 

There are three major computational approaches for drug repositioning: ligand-based, docking-based, and chemogenomic methods [5]. Ligand-based approaches determine the similarity between target proteins’ ligands, in order to predict interactions [5]. Docking-based approaches use the structure of drugs and proteins to compute the interaction likelihood [5]. Chemogenomic approaches include feature-based techniques and similarity-based techniques. Feature-based techniques use features and class labels, and employ machine learning for classification purposes if an input instance corresponds to a positive or negative interaction. In the similarity-based methods, two similarity matrices corresponding to drug and target similarity are used to compute a drug-target interaction matrix [5].

Repositioning existing drugs for new indications is an effective approach used to accelerate the establishment of novel pharmacological treatments for AD patients as the drug candidates have already been through the stages of clinical development and have well-known safety and pharmacokinetic profiles. In the current study, an anti-similarity approach of in silico drug repurposing was employed. Overall, this study aimed at integrating the transcriptional profiles associated with common familial AD mutations in neurons in order to characterize the pathogenetic mechanisms involved in AD and to find more effective drugs for AD.

## 2. Materials and Methods

### 2.1. Dataset Selection and Analysis

The NCBI Gene Expression Omnibus (GEO) database (http://www.ncbi.nlm.nih.gov/geo/ (accessed on 5 December 2021)) was used to identify transcriptomic datasets for the generation of an AD-related signature. The GSE137202 dataset was finally selected as it included whole-genome expression profiles of SH-SY5Y cells, modified to harbor familial AD mutations (APP^V7171F^ and PSEN1^M146V^) [6]. The submitter-supplied data were used for the analysis. Briefly, the dataset was generated using the Affymetrix PrimeView™ Human Gene Expression Arrays and raw data were normalized using the robust multichip analysis (RMA) algorithm [6]. The web-based application ImaGEO was used to perform the meta-analysis (http://bioinfo.genyo.es/imageo/ (accessed on 5 December 2021)) [7]. For the meta-analysis of the AD signature, a random-effects model of effect size measure was used to integrate gene expression patterns (the script employed by ImaGEO is supplied as Supplementary File S1).

Functional enrichment and gene ontology analysis was performed using the web-based software Metascape (accessed on 7 December 2021), using default specifications [8]. Unless otherwise specified, an adjusted (Benjamini–Hochberg-corrected) *p*-value (adj. *p*-value or FDR—false discovery rate) of <0.05 was determined as the threshold for statistical significance.

### 2.2. In Silico Pharmacology

The drug meta-signatures were obtained from Himmelstein et al. [9], which were generated using the Library of Integrated Network-Based Cellular Signatures (LINCS) L1000 perturbation data (http://www.lincsproject.org (accessed on 10 December 2021)) [9]. To date, the L1000 database contains > 40,000 genetic and small molecule perturbations, obtained on a number of established cell lines [10]. Briefly, for the generation of the meta-signatures, the 978 measured landmark genes and the 6489 best-inferred genes were used, and the Stouffer’s meta-analysis method was applied on the z-scores to calculate the consensus drug meta-signature [11]. In the current study, we included only the drugs that received FDA approval. Anti-signature perturbation analysis was performed using the DEGs identified for AD and the drug meta-signatures by using cosine similarity as distance metrics. Ten thousand perturbations were used for the assessment of statistical significance. Hierarchical clustering and similarity matrices were constructed using cosine distance on complete linkage. Analysis was performed using the Morpheus web-based application (https://software.broadinstitute.org/morpheus/, accessed on 15 April 2022). Among the predicted drugs, we identified those with blood–brain barrier (BBB) permeability by interrogating the large benchmark data set, B3DB, which includes 7807 small molecules [12].

## 3. Results

### 3.1. Identification of the AD Gene Expression Profile

The GEO dataset GSE137202 was selected for the determination of the transcriptional profiles that characterize the presence of two familial AD mutations (APP^V7171F^ and PSEN1^M146V^). A total of 641 DEGs were found to be associated with the APP^V7171F^ mutation and 584 DEGs were found to be associated with the PSEN1^M146V^ mutation. Overall, the meta-analysis identified 1479 DEGs—684 downregulated and 795 upregulated. The top 50 DEGs are provided in Table 1.

**Figure 2:**
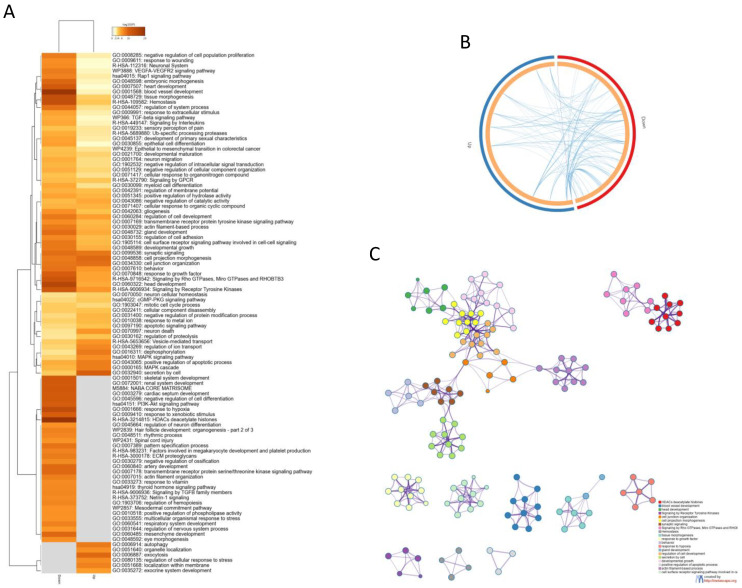
(**A**) Heatmap showing the top 100 enriched terms among the upregulated and downregulated DEGs identified in the meta-analysis; (**B**) Circos plot showing the enriched biological processes overlapping among the up- and downregulated DEGs identified in the meta-analysis; (**C**) network showing the connection among the most enriched pathways by the genes identified in the meta-analysis.

Gene ontology analysis revealed several pathways enriched by the AD DEGs (Figure 2A). A number of enriched processes were enriched by both the up- and downregulated DEGs (Figure 2A,B). The top five most significant enrichment processes were: HDACs deacetylate histones (R-HSA-3214815); blood vessel development (GO:0001568); head development (GO:0060322); signaling by receptor tyrosine kinases (R-HSA-9006934); and cell junction organization (GO:0034330). A network of the connections among the most enriched processes is provided in Figure 2C). Interestingly, HDACs deacetylate histones (R-HSA-3214815) and (GO:0001666) response to hypoxia were the most enriched processes among the downregulated DEGs, while exocytosis (GO:0006887) and autophagy (GO:0006914) hypoxia were the most enriched processes among the upregulated DEGs (Figure 2).

### 3.2. Prediction of Novel Chemotherapeutics for AD

**Figure 3:**
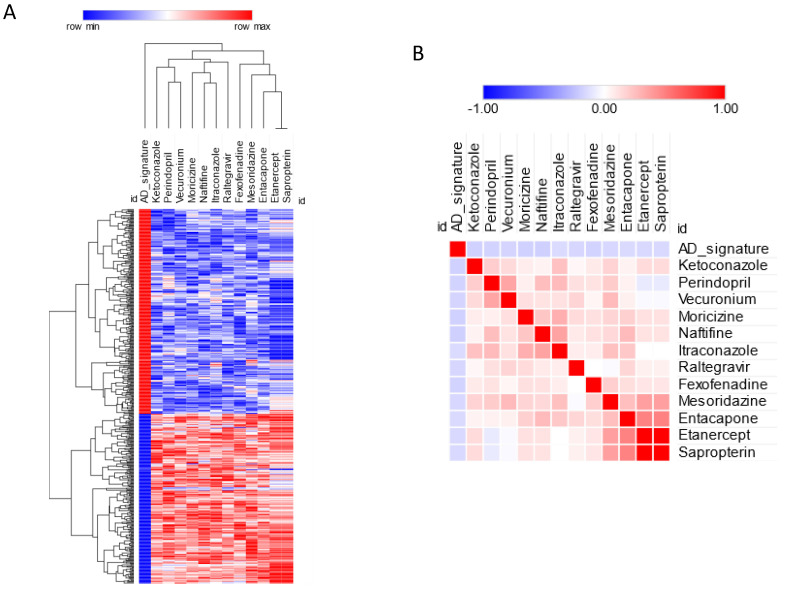
(**A**) Predicted drugs for AD based on anti-similarity; (**B**) similarity matrix for the predicted drugs.

Anti-signature perturbation analysis was performed using the DEGs identified in the meta-analysis and the meta-signature of drugs from the L1000 database. Only the FDA- approved drugs were used for the current analysis. In total, the pairwise similarity was calculated between the AD signature and 752 approved drugs (*p* value distribution is presented as Supplementary Figure S1). Overall, we found 14 drugs with significant anti-similarity to the AD signature (FDR < 0.05) (Figure 3, Table 2). The top five drugs with significant anti-similarity to AD were: naftifine, an anti-mycotic drug; moricizine, used to treat arrhythmias; ketoconazole, an anti-mycotic drug; perindopril, an ACE inhibitor; and fexofenadine, an antihistamine drug (Figure 3, Table 2).

### 3.3. Prediction of Drugs That May Predispose to AD

**Figure 4:**
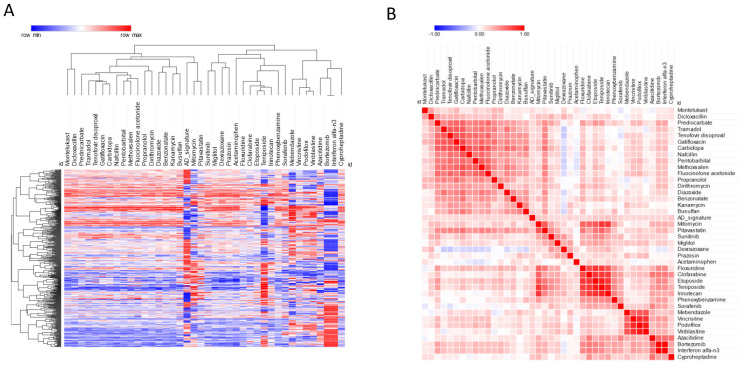
(**A**) Hierarchical clustering for the drugs predicted to predispose to AD; (**B**) similarity matrix for the drugs predicted to predispose to AD.

Among the screened drugs, some showed a transcriptomic profile concordant with that of AD, which may suggest the potential effect of these drugs to potentially induce drug-related AD-like conditions. In particular, we found 39 drugs with a significant concordant signature with the AD profile (FDR < 0.05) (Figure 4, Table 3). The top five drugs in this category were: irinotecan, an anticancer chemotherapeutic; cyproheptadine, an antihistamine; teniposide, an anti-cancer drug; phenoxybenzamine, an alpha-receptor blocking agent used for the treatment of hypertension; and pitavastatin, an HMG-CoA reductase inhibitor (Figure 4, Table 3).

## 4. Discussion

AD, the most common form of age-related dementia, occurs either sporadically or as the early-onset familial form of AD (fAD). Heterozygous germline mutations in either the APP gene or the presenilin (PSEN1 and PSEN2) genes are responsible for fAD. More than 200 fAD mutations in APP, PSEN1, and PSEN2 have been identified, which are responsible for aberrant APP metabolism, with consequent accumulation of abnormal Aβ peptides. This impairs synaptic transmission and causes neurotoxicity. APP duplication or N-terminal mutations lead to an indiscriminate increase in Aβ levels, while mutations at the C-terminal of APP, mostly affecting the γ-secretase site, increase the amount of longer and more hydrophobic Aβ peptides [13]. Mutations in PSEN1 also affect Aβ production [14].

Although the causal role of these mutations in the APP and PSEN1 genes has long been known, their precise consequences at the cellular level remain incompletely characterized, which makes the identification of effective novel therapeutic strategies more challenging. In the present study, by using a meta-analysis approach, we carried out the profiling of mutant cells bearing two fAD mutations (APP^V7171F^ and PSEN1^M146V^) in order to identify commonly perturbed disease-associated transcripts and relevant molecular processes. In silico approaches for the establishment of pathogenetic pathways and for the identification of potential novel pharmacological strategies have largely been employed in recent years by our group and others in a wide range of settings, from cancer to autoimmunity to neurodegeneration [1,15,16,17,18,19,20,21,22,23,24,25].

It is assumed that Aβ plaque accumulation exerts neurotoxicity by hindering the normal synaptic transmission; therefore, the gene expression changes observed in mutant cells likely reflect compensatory feedback aimed at overcoming the effect of pathological Aβ production. However, the neurotoxicity of Aβ plaque is also partially mediated by inflammatory responses, sustained by local microglial cells and astrocytes [26], which further worsens synapse degeneration and neuronal death. Future studies are hence necessary to determine whether the selective targeting of altered pathways of fAD mutant neurons is sufficient for effectively managing the progression of neuropathological changes in AD and the clinical features of the disease.

At present, in silico approaches have largely been exploited for the selection of promising drugs for bench investigations. Drug repurposing, i.e., the use of drugs already approved with different indications, allows us to expedite the search for novel therapeutic treatments [27,28], as the safety and therapeutic range are already known [27,28]. Up to now, the available treatments for AD, such as anticholinesterase inhibitors and N-methyl-D-aspartate receptor antagonists, are able to offer only short-term symptomatic improvement, but cannot inhibit disease progression. Currently, the only target-specific drug, aducanumab, an anti-Aβ monoclonal antibody, first approved by the Food and Drug Administration (FDA) in 2021, has shown limited efficacy and has not received marketing authorization by the European Medicines Agency. Hence, greater efforts are needed to identify more effective therapeutic strategies for better management of AD patients.

Here, we identified potential anti-AD drugs by means of an in silico approach that relies on the anti-similarity between the transcriptional signature of the drugs and the AD-related gene expression profile [29,30,31,32,33]. Among the short-listed drugs, the angiotensin-converting enzyme (ACE) inhibitor, perindopril, was found to be one of the top five predicted drugs. Interestingly, Dong et al. have previously shown that by inhibiting hippocampal ACE, perindropil was able to significantly prevent cognitive impairment in a model of AD induced by intracerebroventricular injection of Aβ_1–40_, as well as in PS2APP-transgenic mice, and was associated with the suppression of microglia and astrocyte activation, and a reduction in oxidative stress [34].

Among the other predicted drugs, fexofenadine was also found to have potential. Fexofenadine is a third-generation antihistamine used for seasonal allergic rhinitis. In a previous study performed on healthy subjects, fexofenadine did not demonstrate impairment of cognitive or psychomotor performance, but positively affected the reaction time for performance of the word memory task [35].

A significant anti-similarity was also observed between the anti-TNF-alpha, etanercept, and the AD signature. Neuroinflammation is a feature of AD brain pathology [36], and the role of the pro-inflammatory cytokine, tumor necrosis factor-alpha (TNF-alpha), in the pathogenesis AD has long been presumed [37,38]. On this basis, a single-center, open-label, pilot (proof-of-concept) study was conducted, which included 15 patients with mild-to-severe AD treated for 6 months with etanercept, 25–50 mg, once weekly, by perispinal administration. In accordance with our findings which suggest the beneficial potential of etanercept, at the end of the treatment schedule, a significant improvement was observed for all the outcome measures, including the Mini-Mental State Examination (MMSE), the Alzheimer’s Disease Assessment Scale—Cognitive Subscale (ADAS-Cog), and the Severe Impairment Battery (SIB) [39]. In any case, early diagnosis and treatment may constitute the optimum strategy for preserving the patient’s quality of life and delaying the development of the disease [40].

It is worth mentioning that some of the identified drugs are not able to cross the BBB. Although this may be a limitation of our study, it should be considered that several biomaterial-based strategies are under development to overcome the BBB and deliver the drug into the brain, such as polymeric nanoparticles, liposomes, and nanogels [41]. Hence, proper delivery strategies are warranted as part of translational drug research for AD.

In our analysis, we also identified drugs whose expression profiles are concordantly modulated to the AD signature. Among them, we found the epigenetic drug, azacytidine. This finding is in accordance with the previous investigation into the role of DNA hypomethylation in AD. Indeed, high levels of S-adenosylhomocysteine, which inhibits DNA methyltransferases, have been found in AD brains and negatively correlate with patient cognitive abilities. Furthermore, DNA hypomethylation of APP, BACE1, and PSEN1 has been observed in the AD brain [42,43].

Moreover, bortezomib was found to induce an expression profile concordant with the AD transcriptomic signature. Bortezomib is a proteasome inhibitor used for the treatment of multiple myeloma. In support of our prediction, it was previously observed in in vitro neuronal models that bortezomib increased the levels of ubiquitin-conjugated proteins and augmented the levels of the pro-apoptotic proteins, PUMA and Noxa. In addition, it increased neuronal cell death, partly via a caspase 3-dependent pathway [44]. We may hence speculate that these drugs may promote the development of AD in susceptible individuals or at least worsen disease progression.

Unexpectedly however, some of the drugs predicted to have a concordant signature with AD have previously been shown to be able to ameliorate the AD condition, as is the case for sorafenib [45] and montelukast [46]. Although this observation requires careful attention and validation in the in vivo setting, we may hypothesize that the pathways targeted by these drugs, rather than being pathological, may instead represent compensatory responses to the concomitant aberrant processes associated with the presence of fAD mutations. Moreover, biomolecular pathways may be differently induced in the different brain cells; hence, the protective effect of such drugs may be related to the targeting of cells other than the neurons (i.e., microglia and astrocyte) which are directly involved in AD pathogenesis.

Still, it is worth emphasizing that while the pathogenetic pathways in AD and the potential therapeutic drugs need to be unequivocally identified, drug treatment supporting psychological interventions, including group activities for patients, should be carefully considered by clinicians as well [47]. Psychological interventions for AD caregivers, such as time-limited group therapy, are also paramount [48,49].

## 5. Conclusions

Overall, this study aimed to integrate the transcriptional profiles associated with common fAD mutations in neurons in order to find more effective drugs for AD. Because of the high number of existing drugs, in silico approaches are a valuable tool for short-listing potential drug candidates to be validated in biological experiments and in patients [36]. There are, however, some limitations in this approach. First, AD is a complex disease with pathological features that arise from the continuous cross-talk among the different brain cell populations, which cannot be fully recapitulated in vitro. Second, the efficacy of a drug is determined by several factors and does not depend on the simple match of expression profiles. Indeed, in the case of AD, drugs have to reach the CNS tissue at the appropriate concentrations in order to exert an effect; hence, the dosage and schedule of administration should be carefully selected and possibly personalized to the patients. On a final note, the drug meta-signatures come from in vitro data generated from established/cancer cell lines that do not fully mimic the central nervous system neural cells. It is also likely that many of the prioritized drugs may have adverse or off-target effects. However, these drugs are in current clinical use and have already been characterized for their pharmacokinetics and toxicity. Despite these limitations, our study sets the basis for future investigations into the pathogenetic processes occurring in AD, and proposes the repurposing of drugs for the treatment of AD to be validated, first in in vivo animal models and subsequently in phase II clinical trials.

## Figures and Tables

**Table 1:** Top 50 modulated genes in AD.

ID	fdr_pval	Pval	zval	Qval	Qpval	Gene_Name
40979	0.0029	4.9 × 10^-6^	−4.6	0.74	0.39	NA
ABCC10	0.0029	5.9 × 10^-6^	4.5	0.22	0.64	ATP-binding cassette subfamily C member 10
ARID3B	0.0029	3.6 × 10^-6^	4.6	0.053	0.82	AT-rich interaction domain 3B
AVEN	0.0029	7.2 × 10^-6^	4.5	0.015	0.9	apoptosis and caspase activation inhibitor
C17orf28	0.0029	4.1 × 10^-6^	4.6	0.014	0.91	NA
C18orf1	0.0029	2.4 × 10^-6^	−4.7	0.06	0.81	NA
C9orf123	0.0029	3.1 × 10^-6^	4.7	0.049	0.82	NA
CALB1	0.0029	3 × 10^-6^	−4.7	0.12	0.73	calbindin 1
CASP9	0.0029	7.3 × 10^-6^	4.5	0.095	0.76	caspase 9
CCDC74B	0.0029	1.9 × 10^-6^	−4.8	0.49	0.48	coiled-coil domain containing 74B
CD9	0.0029	5 × 10^-6^	−4.6	0.078	0.78	CD9 molecule
CMTM7	0.0029	7.5 × 10^-6^	−4.5	0.019	0.89	CKLF-like MARVEL transmembrane domain containing 7
CNTNAP2	0.0029	2.2 × 10^-6^	4.7	0.000046	0.99	contactin-associated protein-like 2
CUX2	0.0029	1.3 × 10^-6^	−4.8	0.2	0.65	cut-like homeobox 2
DNER	0.0029	6.2 × 10^-6^	−4.5	0.48	0.49	delta/notch-like EGF repeat containing
EBF3	0.0029	5.4 × 10^-6^	4.5	1	0.31	early B cell factor 3
EDNRA	0.0029	1.5 × 10^-6^	−4.8	0.083	0.77	endothelin receptor type A
FEZ1	0.0029	7.5 × 10^-6^	−4.5	0.014	0.9	fasciculation and elongation protein zeta 1
FOXD1	0.0029	2.7 × 10^-6^	4.7	0.26	0.61	forkhead box D1
GAS2L3	0.0029	8.1 × 10^-6^	−4.5	0.16	0.69	growth arrest specific 2 like 3
GRIK4	0.0029	5.8 × 10^-6^	−4.5	0.0041	0.95	glutamate ionotropic receptor kainate type subunit 4
GRIP1	0.0029	7.3 × 10^-6^	−4.5	0.2	0.65	glutamate receptor-interacting protein 1
GRM8	0.0029	6.5 × 10^-6^	−4.5	0.5	0.48	glutamate metabotropic receptor 8
HIST1H3F	0.0029	6.4 × 10^-6^	−4.5	0.00057	0.98	histone cluster 1 H3 family member f
HOXA5	0.0029	7.9 × 10^-6^	−4.5	1.1	0.3	homeobox A5
IGF2AS	0.0029	1.8 × 10^-6^	4.8	0.02	0.89	NA
ISLR	0.0029	3.9 × 10^-6^	−4.6	0.17	0.68	immunoglobulin superfamily containing leucine-rich repeat
ITGA2	0.0029	4.7 × 10^-6^	−4.6	0.34	0.56	integrin subunit alpha 2
KAL1	0.0029	5.3 × 10^-6^	4.6	0.45	0.5	NA
KCNC4	0.0029	8 × 10^-6^	4.5	0.78	0.38	potassium voltage-gated channel subfamily C member 4
KCNH2	0.0029	7.2 × 10^-6^	−4.5	0.000082	0.99	potassium voltage-gated channel subfamily H member 2
KIF20A	0.0029	6.6 × 10^-6^	−4.5	0.054	0.82	kinesin family member 20A
LEF1	0.0029	7 × 10^-6^	−4.5	0.95	0.33	lymphoid enhancer-binding factor 1
LHFPL3	0.0029	3.5 × 10^-6^	−4.6	0.68	0.41	LHFPL tetraspan subfamily member 3
LMO2	0.0029	7 × 10^-6^	4.5	0.65	0.42	LIM domain only 2
LOX	0.0029	5.1 × 10^-6^	−4.6	0.075	0.78	lysyl oxidase
NEDD9	0.0029	1.8 × 10^-6^	−4.8	0.0085	0.93	neural precursor cell expressed, developmentally downregulated 9
NEK6	0.0029	6.3 × 10^-6^	−4.5	0.011	0.92	NIMA-related kinase 6
PPEF1	0.0029	6.7 × 10^-6^	−4.5	0.057	0.81	protein phosphatase with EF-hand domain 1
RAP1A	0.0029	1.3 × 10^-6^	4.8	0.00039	0.98	RAP1A, member of the RAS oncogene family
RASL11B	0.0029	8.2 × 10^-6^	−4.5	0.017	0.9	RAS-like family 11 member B
RGS16	0.0029	1.5 × 10^-6^	4.8	0.01	0.92	regulator of G protein signaling 16
RNF152	0.0029	6.9 × 10^-6^	−4.5	0.47	0.49	ring finger protein 152
RUNX1T1	0.0029	2.8 × 10^-6^	4.7	0.073	0.79	RUNX1 translocation partner 1
SERPINF1	0.0029	3.1 × 10^-6^	4.7	0.05	0.82	serpin family F member 1
SIK3	0.0029	3.1 × 10^-6^	4.7	0.25	0.62	SIK family kinase 3
SLIT1	0.0029	5.7 × 10^-6^	−4.5	0.0099	0.92	slit guidance ligand 1
SLIT2	0.0029	2 × 10^-6^	−4.8	0.02	0.89	slit guidance ligand 2
TCEAL2	0.0029	5.8 × 10^-6^	−4.5	0.067	0.8	transcription elongation factor A like 2
TCTA	0.0029	4.1 × 10^-6^	4.6	0.18	0.67	T cell leukemia translocation altered

**Table 2:** Predicted drugs for AD, based on anti-similarity.

ID	Cosine Similarity	FDR(BH)	BBB*
Naftifine	−0.18	0.01	BBB-
Moricizine	−0.18	0.02	BBB-
Ketoconazole	−0.18	0.02	BBB+
Perindopril	−0.17	0.02	BBB-
Fexofenadine	−0.17	0.02	BBB-
Vecuronium	−0.17	0.03	n.a.
Mesoridazine	−0.16	0.02	BBB+
Raltegravir	−0.16	0.03	n.a.
Sapropterin	−0.15	0.03	n.a.
Entacapone	−0.15	0.03	BBB+
Etanercept	−0.15	0.03	n.a.
Trimipramine	−0.14	0.03	BBB+
Trifluoperazine	−0.14	0.04	BBB+
Itraconazole	−0.14	0.04	BBB+

BBB+: permeable to the blood–brain barrier; BBB-: not permeable to the blood–brain barrier; n.a.: not available.

**Table 3:** Predicted drugs predisposed to AD.

ID	Cosine Similarity	FDR(BH)	BBB
Irinotecan	0.24	0.01	BBB-
Cyproheptadine	0.22	0.01	BBB+
Teniposide	0.22	0.01	BBB+
Phenoxybenzamine	0.22	0.01	BBB-
Pitavastatin	0.22	0.01	n.a.
Mitomycin	0.21	0.01	BBB-
Etoposide	0.21	0.01	BBB-
Busulfan	0.19	0.02	BBB+
Sorafenib	0.18	0.01	BBB+
Prazosin	0.18	0.02	BBB+
Fluocinolone acetonide	0.18	0.02	n.a.
Dirithromycin	0.17	0.01	BBB-
Bortezomib	0.17	0.02	n.a.
Podofilox	0.17	0.02	n.a.
Interferon alfa-n3	0.17	0.02	n.a.
Vinblastine	0.17	0.03	BBB+
Carbidopa	0.16	0.02	BBB-
Pentobarbital	0.16	0.02	BBB+
Acetaminophen	0.16	0.02	n.a.
Vincristine	0.16	0.02	BBB-
Methoxsalen	0.16	0.02	BBB-
Propranolol	0.16	0.02	BBB-
Clofarabine	0.16	0.02	BBB-
Gatifloxacin	0.16	0.02	BBB-
Mebendazole	0.16	0.03	BBB-
Benzonatate	0.16	0.03	BBB-
Azacitidine	0.16	0.03	n.a.
Dicloxacillin	0.15	0.02	BBB-
Tenofovir disoproxil	0.15	0.03	n.a.
Floxuridine	0.15	0.03	BBB-
Miglitol	0.15	0.03	BBB-
Diazoxide	0.15	0.03	BBB-
Bupropion	0.15	0.03	BBB+
Dexrazoxane	0.15	0.04	BBB-
Kanamycin	0.15	0.04	BBB-
Montelukast	0.14	0.03	n.a.
Nafcillin	0.14	0.03	BBB-
Sunitinib	0.14	0.03	BBB+
Tramadol	0.14	0.04	BBB+
Cephalexin	0.14	0.04	BBB-
Prednicarbate	0.14	0.04	BBB+
Clopidogrel	0.13	0.03	BBB-

BBB+: permeable to the blood–brain barrier; BBB-: not permeable to the blood–brain barrier; n.a.: not available.

## Data Availability

Data are generated using the GSE137202 dataset, freely-available from the gene expression Omnibus database (https://www.ncbi.nlm.nih.gov/gds (accessed on 5 December 2021)).

## Supplementary Materials

The following supporting information can be downloaded at: https://www.mdpi.com/article/10.3390/brainsci12070827/s1.

## References

[1] Cavalli E., Battaglia G., Basile M.S., Bruno V., Petralia M.C., Lombardo S.D., Pennisi M., Kalfin R., Tancheva L., Fagone P. (2020). Exploratory Analysis of iPSCS-Derived Neuronal Cells as Predictors of Diagnosis and Treatment of Alzheimer Disease. Brain Sci..

[2] Rujeedawa T., Félez E.C., Clare I.C.H., Fortea J., Strydom A., Rebillat A.-S., Coppus A., Levin J., Zaman S.H. (2021). The Clinical and Neuropathological Features of Sporadic (Late-Onset) and Genetic Forms of Alzheimer’s Disease. J. Clin. Med..

[3] Wu M., Zhang M., Yin X., Chen K., Hu Z., Zhou Q., Cao X., Chen Z., Liu D. (2021). The role of pathological tau in synaptic dysfunction in Alzheimer’s diseases. Transl. Neurodegener..

[4] Lichtenthaler S.F., Tschirner S.K., Steiner H. (2021). Secretases in Alzheimer’s disease: Novel insights into proteolysis of APP and TREM2. Curr. Opin. Neurobiol..

[5] Mongia A., Majumdar A. (2020). Drug-target interaction prediction using Multi Graph Regularized Nuclear Norm Minimization. PLoS ONE.

[6] Duran-Frigola M., Pauls E., Guitart-Pla O., Bertoni M., Alcalde V., Amat D., Juan-Blanco T., Aloy P. (2020). Extending the small-molecule similarity principle to all levels of biology with the Chemical Checker. Nat. Biotechnol..

[7] Toro-Domínguez D., Martorell-Marugán J., López-Domínguez R., García-Moreno A., González-Rumayor V., Alarcón-Riquelme M.E., Carmona-Sáez P. (2019). ImaGEO: Integrative gene expression meta-analysis from GEO database. Bioinformatics.

[8] Zhou Y., Zhou B., Pache L., Chang M., Khodabakhshi A.H., Tanaseichuk O., Benner C., Chanda S.K. (2019). Metascape provides a biologist-oriented resource for the analysis of systems-level datasets. Nat. Commun..

[9] Keenan A.B., Jenkins S.L., Jagodnik K.M., Koplev S., He E., Torre D., Wang Z., Dohlman A.B., Silverstein M.C., Lachmann A. (2018). The Library of Integrated Network-Based Cellular Signatures NIH Program: System-Level Cataloging of Human Cells Response to Perturbations. Cell Syst..

[10] Subramanian A., Narayan R., Corsello S.M., Peck D.D., Natoli T.E., Lu X., Gould J., Davis J.F., Tubelli A.A., Asiedu J.K. (2017). A next generation connectivity map: L1000 platform and the first 1,000,000 profiles. Cell.

[11] Himmelstein D.S., Lizee A., Hessler C., Brueggeman L., Chen S.L., Hadley D., Green A., Khankhanian P., Baranzini S.E. (2017). Systematic integration of biomedical knowledge prioritizes drugs for repurposing. eLife.

[12] Meng F., Xi Y., Huang J., Ayers P.W. (2021). A curated diverse molecular database of blood-brain barrier permeability with chemical descriptors. Sci. Data.

[13] Herl L., Thomas A.V., Lill C.M., Banks M., Deng A., Jones P.B., Spoelgen R., Hyman B.T., Berezovska O. (2009). Mutations in amyloid precursor protein affect its interactions with presenilin/γ-secretase. Mol. Cell. Neurosci..

[14] Shen J., Kelleher R.J. (2007). The presenilin hypothesis of Alzheimer’s disease: Evidence for a loss-of-function pathogenic mechanism. Proc. Natl. Acad. Sci. USA.

[15] Świetlik D., Białowąs J., Kusiak A., Krasny M. (2022). Virtual Therapy with the NMDA Antagonist Memantine in Hippocampal Models of Moderate to Severe Alzheimer’s Disease, in Silico Trials. Pharmaceuticals.

[16] Świetlik D., Kusiak A., Ossowska A. (2022). Computational Modeling of Therapy with the NMDA Antagonist in Neurodegenerative Disease: Information Theory in the Mechanism of Action of Memantine. Int. J. Environ. Res. Public Health.

[17] Świetlik D., Kusiak A., Krasny M., Białowąs J. (2022). The Computer Simulation of Therapy with the NMDA Antagonist in Excitotoxic Neurodegeneration in an Alzheimer’s Disease-like Pathology. J. Clin. Med..

[18] Fagone P., Mangano K., Martino G., Quattropani M.C., Pennisi M., Bella R., Fisicaro F., Nicoletti F., Petralia M.C. (2022). Characterization of Altered Molecular Pathways in the Entorhinal Cortex of Alzheimer’s Disease Patients and In Silico Prediction of Potential Repurposable Drugs. Genes.

[19] Mammana S., Bramanti P., Mazzon E., Cavalli E., Basile M.S., Fagone P., Petralia M.C., McCubrey J.A., Nicoletti F., Mangano K. (2018). Preclinical evaluation of the PI3K/Akt/mTOR pathway in animal models of multiple sclerosis. Oncotarget.

[20] Mangano K., Cavalli E., Mammana S., Basile M.S., Caltabiano R., Pesce A., Puleo S., Atanasov A., Magro G., Nicoletti F. (2017). Involvement of the Nrf2/HO—1/CO axis and therapeutic intervention with the CO—releasing molecule CORM—A1, in a murine model of autoimmune hepatitis. J. Cell. Physiol..

[21] Basile M.S., Fagone P., Mangano K., Mammana S., Magro G., Salvatorelli L., Destri G.L., La Greca G., Nicoletti F., Puleo S. (2019). KCNMA1 Expression Is Downregulated in Colorectal Cancer via Epigenetic Mechanisms. Cancers.

[22] Fagone P., Ciurleo R., Lombardo S.D., Iacobello C., Palermo C.I., Shoenfeld Y., Bendtzen K., Bramanti P., Nicoletti F. (2020). Transcriptional landscape of SARS-CoV-2 infection dismantles pathogenic pathways activated by the virus, proposes unique sex-specific differences and predicts tailored therapeutic strategies. Autoimmun. Rev..

[23] Cavalli E., Petralia M.C., Basile M.S., Bramanti A., Bramanti P., Nicoletti F., Spandidos D.A., Shoenfeld Y., Fagone P. (2020). Transcriptomic analysis of COVID-19 lungs and bronchoalveolar lavage fluid samples reveals predominant B cell activation responses to infection. Int. J. Mol. Med..

[24] Lombardo S.D., Mazzon E., Mangano K., Basile M.S., Cavalli E., Mammana S., Fagone P., Nicoletti F., Petralia M.C. (2019). Transcriptomic Analysis Reveals Involvement of the Macrophage Migration Inhibitory Factor Gene Network in Duchenne Muscular Dystrophy. Genes.

[25] Lombardo S., Basile M., Ciurleo R., Bramanti A., Arcidiacono A., Mangano K., Bramanti P., Nicoletti F., Fagone P. (2021). A Network Medicine Approach for Drug Repurposing in Duchenne Muscular Dystrophy. Genes.

[26] Hong S., Beja-Glasser V.F., Nfonoyim B.M., Frouin A., Li S., Ramakrishnan S., Merry K.M., Shi Q., Rosenthal A., Barres B.A. (2016). Complement and microglia mediate early synapse loss in Alzheimer mouse models. Science.

[27] Plenge R.M., Scolnick E.M., Altshuler D. (2013). Validating therapeutic targets through human genetics. Nat. Rev. Drug Discov..

[28] Scannell J.W., Blanckley A., Boldon H., Warrington B. (2012). Diagnosing the decline in pharmaceutical R&D efficiency. Nat. Rev. Drug Discov..

[29] Shim J.S., Liu J.O. (2014). Recent Advances in Drug Repositioning for the Discovery of New Anticancer Drugs. Int. J. Biol. Sci..

[30] Liu Z., Fang H., Reagan K., Xu X., Mendrick D.L., Slikker W., Tong W. (2013). In silico drug repositioning—What we need to know. Drug Discov. Today.

[31] Hodos R.A., Kidd B.A., Shameer K., Readhead B.P., Dudley J.T. (2016). In silico methods for drug repurposing and pharmacology. WIREs Syst. Biol. Med..

[32] Jin G., Wong S.T. (2013). Toward better drug repositioning: Prioritizing and integrating existing methods into efficient pipelines. Drug Discov. Today.

[33] Jadamba E., Shin M. (2016). A Systematic Framework for Drug Repositioning from Integrated Omics and Drug Phenotype Profiles Using Pathway-Drug Network. BioMed Res. Int..

[34] Dong Y., Kataoka K., Tokutomi Y., Nako H., Nakamura T., Toyama K., Sueta D., Koibuchi N., Yamamoto E., Ogawa H. (2011). Perindopril, a centrally active angiotensin-converting enzyme inhibitor, prevents cognitive impairment in mouse models of Alzheimer’s disease. FASEB J..

[35] Zannat R., Uddin M.M.N., Rahman A., Aklima J., Al Amin M. (2016). Antihistamines considerably modulate the cognitive and psychomotor performance of human volunteers. Cogent Psychol..

[36] Akiyama H., Barger S., Barnum S., Bradt B., Bauer J., Cole G.M., Cooper N.R., Eikelenboom P., Emmerling M., Fiebich B.L. (2000). Inflammation and Alzheimer’s disease. Neurobiol. Aging.

[37] Tarkowski E., Andreasen N., Blennow K. (2003). Intrathecal inflammation precedes development of Alzheimer’s disease. J. Neurol. Neurosurg. Psychiatry.

[38] Tarkowski E., Liljeroth A.-M., Minthon L., Tarkowski A., Wallin A., Blennow K. (2003). Cerebral pattern of pro- and anti-inflammatory cytokines in dementias. Brain Res. Bull..

[39] Tobinick E. (2007). Perispinal Etanercept for Treatment of Alzheimers Disease. Curr. Alzheimer Res..

[40] Rasmussen J., Langerman H. (2019). Alzheimer’s Disease—Why We Need Early Diagnosis. Degener. Neurol. Neuromuscul. Dis..

[41] Goyal K., Koul V., Singh Y., Anand A. (2014). Targeted drug delivery to central nervous system (CNS) for the treatment of neurodegenerative disorders: Trends and advances. Cent. Nerv. Syst. Agents Med. Chem..

[42] Sung H.Y., Choi E.N., Jo S.A., Oh S., Ahn J.-H. (2011). Amyloid protein-mediated differential DNA methylation status regulates gene expression in Alzheimer’s disease model cell line. Biochem. Biophys. Res. Commun..

[43] Hou Y., Wang F., Cheng L., Luo T., Xu J., Wang H. (2016). Expression Profiles of SIRT1 and APP Genes in Human Neuroblastoma SK-N-SH Cells Treated with Two Epigenetic Agents. Neurosci. Bull..

[44] Pilchova I., Klacanova K., Dibdiakova K., Saksonova S., Stefanikova A., Vidomanova E., Lichardusova L., Hatok J., Racay P. (2017). Proteasome Stress Triggers Death of SH-SY5Y and T98G Cells via Different Cellular Mechanisms. Neurochem. Res..

[45] Kim J., Park J.-H., Park S.K., Hoe H.-S. (2021). Sorafenib Modulates the LPS- and Aβ-Induced Neuroinflammatory Response in Cells, Wild-Type Mice, and 5xFAD Mice. Front. Immunol..

[46] Xiong L.Y., Ouk M., Wu C.-Y., Rabin J.S., Lanctôt K.L., Herrmann N., Black S.E., Edwards J.D., Swardfager W. (2021). Leukotriene receptor antagonist use and cognitive decline in normal cognition, mild cognitive impairment, and Alzheimer’s dementia. Alzheimer’s Res. Ther..

[47] McDermott O., Charlesworth G., Hogervorst E., Stoner C., Moniz-Cook E., Spector A., Csipke E., Orrell M. (2018). Psychosocial interventions for people with dementia: A synthesis of systematic reviews. Aging Ment. Health.

[48] Cheng S.-T., Lau B.R.W.L., Mak B.E.P.M., Ng B.N.S.S., Lam L.C.W. (2014). Benefit-Finding Intervention for Alzheimer Caregivers: Conceptual Framework, Implementation Issues, and Preliminary Efficacy. Gerontologist.

[49] Lenzo V., Gargano M.T., Mucciardi M., Verso G.L., Quattropani M.C. (2014). Clinical Efficacy and Therapeutic Alliance in a Time-Limited Group Therapy for Young Adults. Res. Psychother. Psychopathol. Process Outcome.

