# Supplementary figures and images for: Computational Analysis of Pathogenetic Pathways in Alzheimer’s Disease and Prediction of Potential Therapeutic Drugs
Source: Brain Sci. 2022 Jun 24;12(7):827. doi: 10.3390/brainsci12070827 (PMC9313152; doi:10.3390/brainsci12070827)

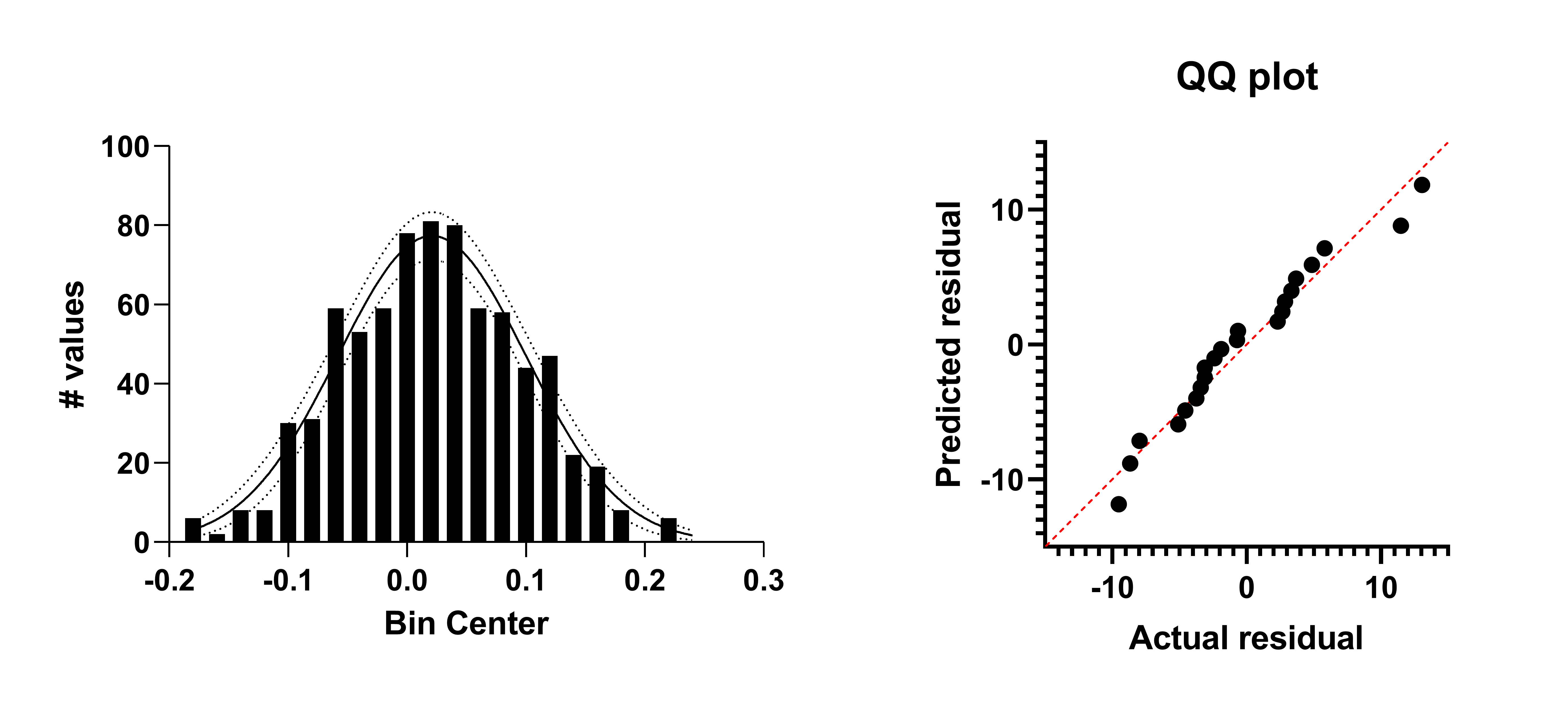

Supplement: Supplementary file 1 [file brainsci-12-00827-s001.zip › suppl material/Supplementary Figure S1.tif]
